# Sustained reduction in numbers of Australian fur seal pups: Implications for future population monitoring

**DOI:** 10.1371/journal.pone.0265610

**Published:** 2022-03-18

**Authors:** Rebecca R. McIntosh, Karina J. Sorrell, Sam Thalmann, Anthony Mitchell, Rachael Gray, Harley Schinagl, John P. Y. Arnould, Peter Dann, Roger Kirkwood

**Affiliations:** 1 Conservation Department, Phillip Island Nature Parks, Cowes, Victoria, Australia; 2 School of Biological Sciences, Monash University, Clayton, Victoria, Australia; 3 Department of Natural Resources and Environment, Hobart, Tasmania, Australia; 4 Department of Environment, Land, Water and Planning, Orbost, Victoria, Australia; 5 Sydney School of Veterinary Science, Faculty of Science, The University of Sydney, New South Wales, Australia; 6 School of Biological and Chemical Sciences, Deakin University, Burwood, Victoria, Australia; 7 South Australian Research and Development Institute—Aquatic Sciences, West Beach, South Australia, Australia; MARE – Marine and Environmental Sciences Centre, PORTUGAL

## Abstract

Fur seal populations in the Southern Hemisphere were plundered in the late 1700s and early 1800s to provide fur for a clothing industry. Millions of seals were killed resulting in potentially major ecosystem changes across the Southern Hemisphere, the consequences of which are unknown today. Following more than a century of population suppression, partly through on-going harvesting, many of the fur seal populations started to recover in the late 1900s. Australian fur seals (*Arctocephalus pusillus doriferus*), one of the most geographically constrained fur seal species, followed this trend. From the 1940s to 1986, pup production remained at approximately 10,000 per year, then significant growth commenced. By 2007, live pup abundance had recovered to approximately 21,400 per year and recovery was expected to continue However, a species-wide survey in 2013 recorded a 20% decline, to approximately 16,500 live pups. It was not known if this decline was due to 2013 being a poor breeding year or a true population reduction. Here we report the results of a population-wide survey conducted in 2017 and annual monitoring at the most productive colony, Seal Rocks, Victoria that recorded a large decline in live pup abundance (-28%). Sustained lower pup numbers at Seal Rocks from annual counts between 2012–2017 (mean = 2908 ± 372 SD), as well as the population-wide estimate of 16,903 live pups in 2017, suggest that the pup numbers for the total population have remained at the lower level observed in 2013 and that the 5-yearly census results are not anomalies or representative of poor breeding seasons. Potential reasons for the decline, which did not occur range-wide but predominantly in the most populated and long-standing breeding sites, are discussed. To enhance adaptive management of this species, methods for future monitoring of the population are also presented. Australian fur seals occupy several distinct regions influenced by different currents and upwellings: range-wide pup abundance monitoring enables comparisons of ecosystem status across these regions. Forces driving change in Australian fur seal pup numbers are likely to play across other marine ecosystems, particularly in the Southern Hemisphere where most fur seals live.

## Introduction

Wildlife populations inhabit continually changing environments that influence population dynamics at small to large spatial scales [1]. By investigating population trends locally as well as across a species’ geographical range, inference can be made on drivers of change, which can assist management decision making (e.g. [2]). Reliable monitoring programs underpin effective management and industry decisions; programs need to be practical, given logistic and resource constraints, and adaptive to accommodate changes in technology, population trends and increased knowledge of drivers [3, 4].

Over the last 200 years, human population growth, colonialism and development have resulted in industrialisation including large-scale harvesting of species, urbanisation and climate change that have modified ecosystems and reduced species diversity [5–8]. Adding to these challenges is the shifting baseline syndrome caused by a lack of historic data that reduces our understanding of pre-colonial ecosystem function [9, 10]. For example: a population could be considered abundant and an ecosystem sustainable because it has increased or sustained relative to its recently recorded history; this view excludes the possibility that the earlier population size may have been larger, and the ecosystem more complex. Shifting baseline syndrome can result in poor management decisions and continued loss of biodiversity in both terrestrial and marine systems.

Australian fur seals (*Arctocephalus pusillus doriferus*), endemic to south-eastern Australia, provide a suitable case study for the value of long-term monitoring, the importance of critical review and adaptation in a monitoring program, and how shifting baseline syndrome can negatively affect conservation. Fur seals are useful indicators of change in marine ecosystems because their health and demographic parameters respond to their environment, they shape marine food webs and are affected by anthropogenic impacts. Foraging occurs at sea while breeding occurs on land during annual, synchronised seasons; with females as central-place foragers, returning regularly to feed pups [11]. These ocean-land characteristics facilitate more detailed research of multiple indicators of change compared to pelagic or migratory species such as sharks and whales that are more logistically difficult to study. However, relying on both domains also exposes fur seals to more threats across coastal and marine regions [12–14].

**Fig 1:**
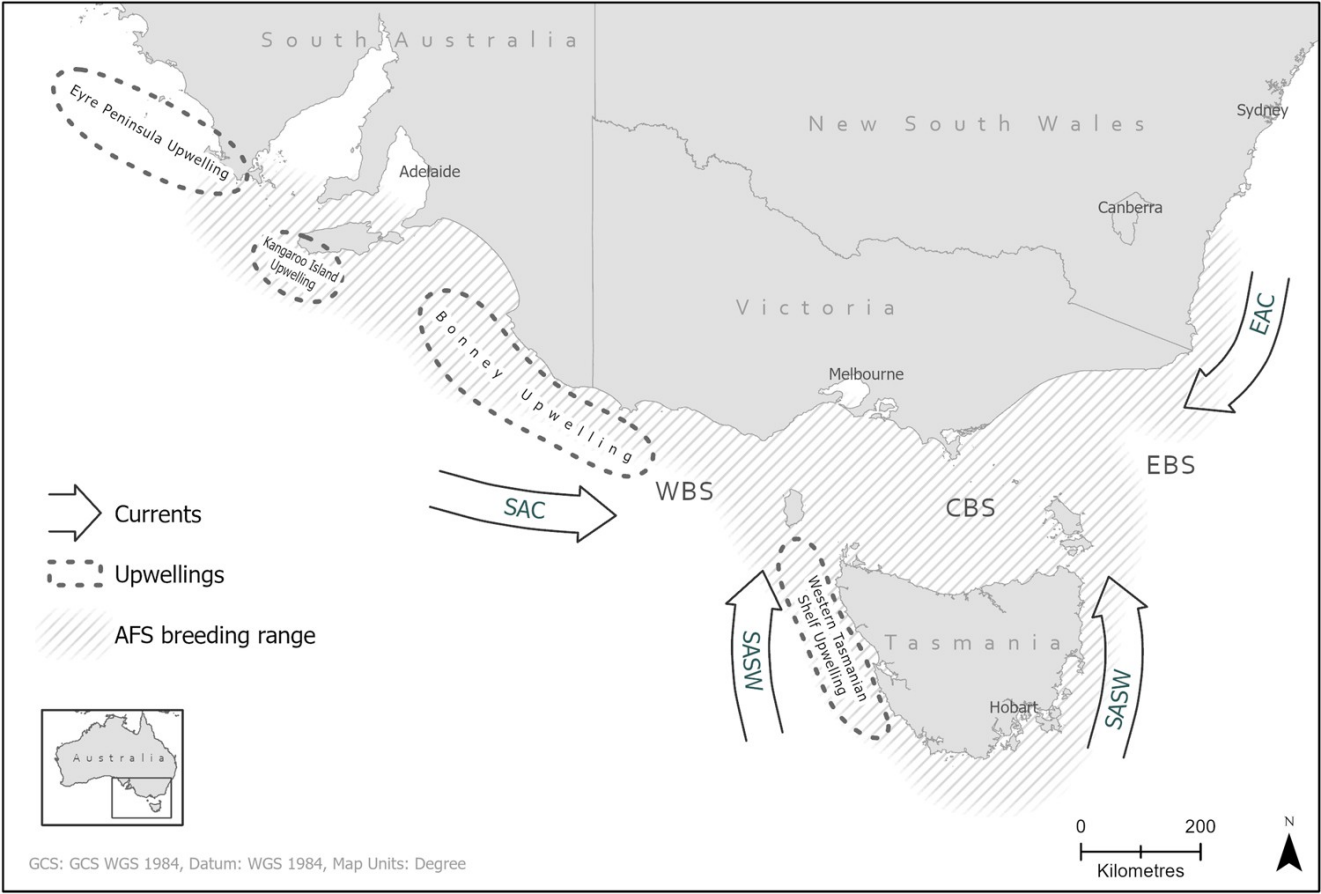
Map of south-eastern Australia showing the breeding range of the Australian fur seal (shaded) [15] and the regional oceanographic influences (currents and cold-water upwellings) [16–18]. Currents and regions are shown by their acronyms: South Australian Current (SAC), Sub-Antarctic Surface Waters (SASW), East Australian Current (EAC), Western Bass Strait (WBS), Central Bass Strait and Eastern Bass Strait (EBS). Geopolitical states (South Australia, Victoria, New South Wales and Tasmania) and associated capital cities are provided for reference. The data in this map is from Geoscience Australia [19].

The Australian fur seal ranges from New South Wales to South Australia, and Tasmania, with the largest breeding colonies occurring in Victorian Bass Strait and the largest productive breeding colony of Seal Rocks, being close to Melbourne, the capital city of Victoria [15] (Fig 1). Breeding colonies occupy distinct oceanic regions influenced by currents and upwelling, i.e., the East Australia Current, Bass Strait, sub-Antarctic Surface waters (around southern Tasmania), West Tasmanian Upwelling Zone and the Bonney Upwelling Zone [16–18].

Australian fur seals respond to their local environment and oceanographic influences and are also subjected to natural predation from white sharks (C*archaradon carcharias*) and orcas (*Orcinus orca*). However, it is human activities that pose the greatest risk to population recovery. Fisheries interactions including incidental mortality in active fishing gear, fish aquaculture interactions, and entanglement in recreational and commercial fishing materials, constitute the most obvious and immediate threat to the species [20]. Marine oil spills from shipping have had devastating impacts for fur seals in the past [21] and recently, pups have been identified with high levels of toxic compounds including persistent organic pollutants such as per- and polyfluoroalkyl substances (PFAS) [22] In the future, rising sea levels and increased storm frequency induced by climate change will threaten many breeding areas [23] because pups are born close to sea level on rocky ledges and at the base of cliffs (Table 1) [15, 24]. Threatening processes that limit population recovery can be synergistic and/or vary across the range of the species; identifying and mitigating these depends upon monitoring the trends of the population reliably.

**Table 1:** Descriptions of colonies (n = 21) visited during the 2017 census of the Australian fur seal and dates of pup estimates from December 2017 to February 2018. Responsible agencies include Phillip Island Nature Parks (PINP), the Department of Land, Water and Planning Victoria (DELWP) and the Department of Nature Resources and Environment Tasmania (NRE).

Colony	Agency	Latitude	Longitude	Area (ha)	Height (m)	Breeding area description	Estimate	Date of pup estimate
**Victoria**								
Deen Maar Island (DMI)	PINP & DELWP	38°25’S	142°00’E	150	40	Inter-tidal platforms, cobble beaches and caves	CMR	7–8 Jan 2018
Seal Rocks (SR)	PINP	38°30’S	145°10’E	8	10	Cobble beaches and outcrop	CMR	28–29 Dec 2017
Kanowna Island (Kan)	Deakin University	39°10’S	146°18’E	130	90	Granite slopes and boulders	Count	16 Dec 2017
The Skerries (Ske)	PINP & DELWP	37°45’S	149°31’E	8	10	Boulder outcrop, three islets	CMR	20 Jan 2018
Rag Island (Rag)	PINP	38°58’S	146°42’E	3	15	Granite slopes and boulders	Count	26 Jan 2018
Cape Bridgewater (CB)	PINP & DELWP	38°23’S	141°24’E	1	0	Cave and inter-tidal platforms	Count	10 Jan 2018
Marengo Reef (MarR)	PINP & DELWP	38°46’S	143°67’E	1	0	Small rocky reef close to shore	Count	4 Jan 2018
**Tasmania**								
Reid Rocks (RR)	NRE	40°14’S	144°09’E	10	8	Series of flat-topped, columnar-dolerite islets	Aerial	18 Jan 2018
West Moncoeur (WM)	NRE	39°14’S	146°30’E	4	30	Steep granite slopes and boulders	NA	NA
Judgment Rocks (JR)	NRE	39°30’S	147°07’E	14	50	Dome shaped, steep, granite, some flat areas	CMR	10 Jan 2018
Tenth Island (TI)	NRE	40°57’S	146°59’E	1	8	Single, low basalt islet	CMR	6 Jan 2018
Moriarty Rocks (MR)	NRE	40°35’S	148°16’E	4	7	Granite islets (East & West)	Aerial	12 Jan 2018
Wright Rocks (WR)	NRE	39°36’S	147°33’E	4	30	Dome shaped, steep, granite	Count	8 Jan 2018
Double Rocks (DR)	NRE	40°20’S	147°55’E	1	15	Flat, rectangular, granite	Count	8 Jan 2018
Bull Rocks (BR)	NRE	40°44’S	147°17’E	1	5	Columnar jointed basalt	Count	5 Jan 2018
Sloop Rocks (Sloop)	NRE	42°18’S	145°10’E	2	15	Granite islets, slopes and boulders	Count	21 Feb 2018
Iles des Phoques (IdP)	NRE	42°25’S	148°09’E	8	7	Granite island	Count	23 Jan 2018
Maatsuyker Is (Maat)	NRE	43°38’S	146°17E	186	284	Quartzite	Count	7 Feb 2018
Wendar Is (Wen)	NRE	43°24’S	145°55’E	5.8	40	Quartzite	Count	6 Feb 2018
Needle Rocks (Nde)	NRE	43°39’S	146°15’E	10.5	42	Quartzite	Count	7 Feb 2018
Walker Island (Wal)	NRE	43°37’S	146°16’E	15	84	Quartzite	Count	7 Feb 2018

**Fig 2:**
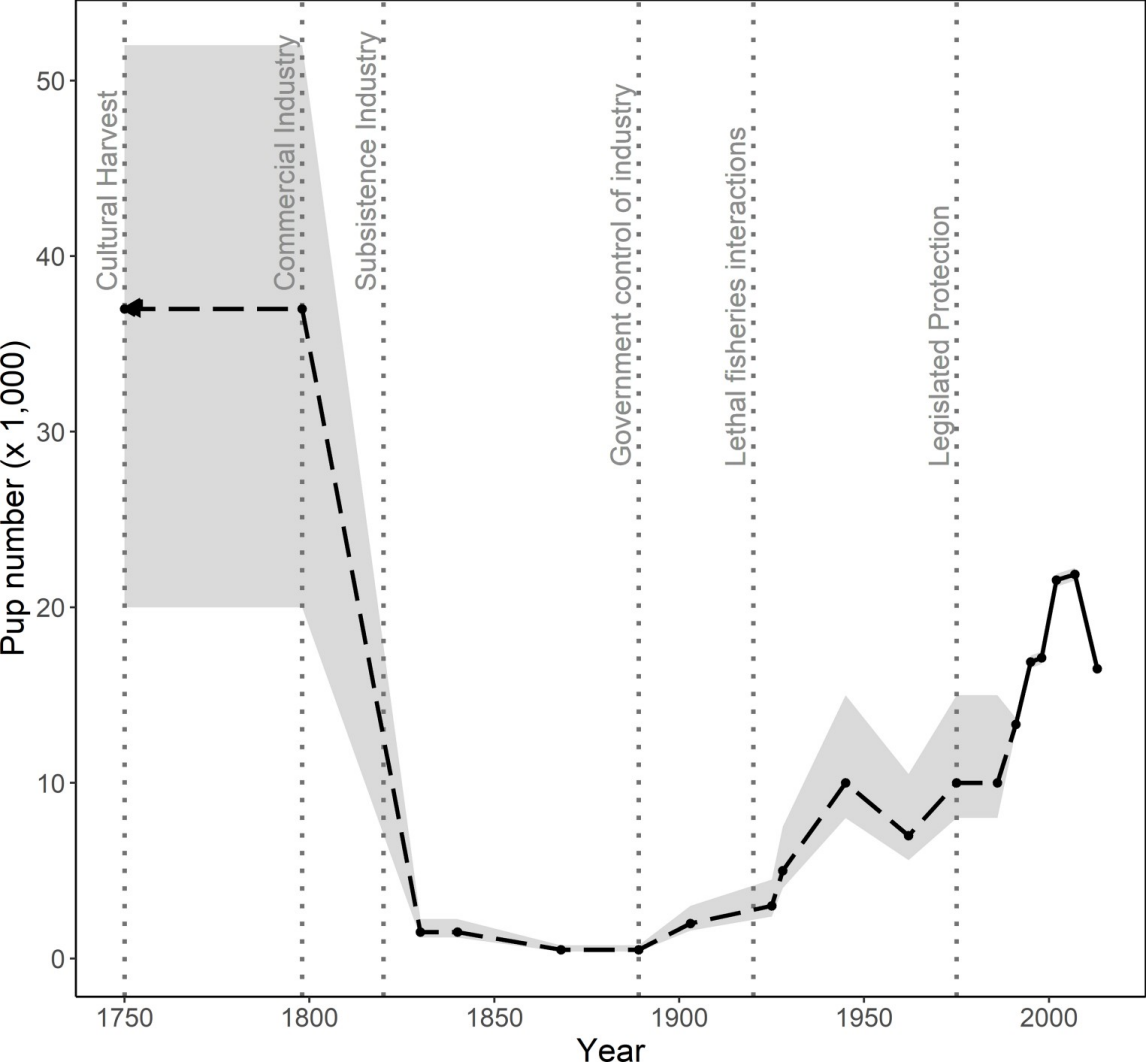
Estimated change in pup numbers of Australian fur seals from 1750 to 2013 (dashed and solid line), assuming thousands of years stability during hunting and cultural use by Aboriginal peoples (Cultural Harvest). The dashed line indicates high levels of uncertainty in the data, the solid line high reliability. Key anthropogenic influences are marked by the dotted vertical lines; the left arrow identifies continuity. Harvesting by commercial sealers began in the late 1700s (Commercial Industry) and declined rapidly to a subsistence industry (Subsistence Industry) when the government protected the seals to some degree and restricted harvesting (Government control of industry). Legal culls and lethal killings by fishers (Lethal fisheries interactions) limited population growth until legislated protection (Legislated Protection) of fur seals in 1975. The shaded ribbon estimates the upper and lower confidence levels for each estimated datapoint, which has improved with standardised and synchronised censuses and represent 95% confidence intervals from 1991–2013. Data and sources provided in (S1 Table in S1 File).

Australian fur seal populations have experienced rapid change over the last 200 years and currently show detectable responses to ecosystem change in their diet, movement, health, and population size [15, 25–27]. Sealing gangs were active in Bass Strait in the late 1700s and by 1830, had driven Australian seal species almost to extinction in just 40 years [28, 29] (Fig 2; S1 Table in S1 File). Suppression of the remaining populations in southern Australia continued as a consequence of uncontrolled fisheries interactions until they received legislative protection in Victoria in 1975 [30, 31]. Then, their populations increased consistently through the 1980s and 1990s. Toward the early 2000s, growth rates in some of the larger populations slowed. However, in 2013, rather than observing a plateau in numbers or continued growth, a drop of 20% in pup abundance was recorded [15] (Fig 2; S1 Table in S1 File). The overall decrease in 2013, the first observed since the recording of pup numbers began in 1986, may have represented an anomalously poor pupping season, or a more sustained reduction but the low frequency of surveys (aimed for every 5 years) reduced the ability to interpret the change [15]. The Australian fur seal is currently listed as of *Least Concern* by the International Union for the Conservation of Nature (IUCN), but recent reductions in pup abundance are concerning.

The lack of clarity regarding the pre-colonial population size and geographical range challenges our ability to contextualise this decline and determine our level of concern. Sealing records have been used to reconstruct an estimate of pre-colonial population size, but the uncertainly is large (Fig 2). Despite this uncertainly, the ecosystem prior to colonisation had greater complexity. For example, the low genetic divergence between the Australian fur seal and the Cape fur seal (*A*. *p*. *pusillus*), suggests a recent dispersal to Australia during the late Pleistocene ~12,000 years ago. On their arrival, they had to coexist with three established seal species (Long-nosed fur seal *Arctocephalus forsteri*, Australian sea lion *Neophoca cinerea* and Southern elephant seal *Mirounga leonina*) [32–34]. Also, seals or *Mering-mum* in the language of the Boon Wurrung peoples of the Kulin Nation [35], were hunted by Aboriginal women in Bass Strait and have been a traditional food source for at least 8,000 years, highlighting the long-term availability of seals in the pre-colonial environment [34, 36]. It is therefore reasonable to assume that at its recent peak in 2007, the fur seal population in Bass Strait may have reached half of the original population size (Fig 2). This is important because the community perspective is that the fur seal population is large and healthy. It is vital to determine whether the 2013 reduction in pup abundance is ongoing to ensure appropriate management responses and because anthropogenic processes that could be affecting the fur seals could have flow-on effects to the marine ecosystem as a whole.

Despite the overall reduction in pup numbers between 2007 and 2013, new smaller also established, particularly around the periphery of the range [15, 24, 37]. This is not unexpected because the fur seals have been expanding their range over recent decades. Also, the individual colonies across the range form a metapopulation, where local populations are relatively discrete spatial entities, but they interact, and some migration is expected evidenced by substantial gene flow and low genetic diversity [33], as well as tracking data that shows seals visiting multiple sites across the range [38]. Under the metapopulation paradigm [39], animal movement between individual colonies should maintain a balance between them; local declines may occur as other sites increase and there are unoccupied sites that may be colonised. However, sudden local declines of previously stable or growing populations can indicate widespread issues with metapopulation health [40]. The pup trends observed in 2013 for the Australian fur seal metapopulation could indicate migration and redistribution; however, the number of births at new sites did not balance the loss of pups at the established sites, and as such, other factors may be influencing pup production and/or pup survival [15].

In wildlife management, range-wide estimates provide metapopulation data necessary for ecosystem modelling and planning, detecting range expansions and new breeding sites. Being logistically complex in a time of funding constraints, species-wide pup production estimates for Australian fur seals in recent years have been performed every five years (2002, 2007, and 2013) [15, 24, 37]. While enabling broad changes in abundance and range to be assessed, the 5-yearly gap between estimates resulted in low confidence of detecting and interpreting population change [15]. To augment the range-wide census, intensive programs (including focussed research and annual pup surveys) are performed at selected sites, enhancing the confidence in the 5-yearly metapopulation trend and improving our understanding of processes and potential factors driving population change [27, 41–44].

Our study aims to clarify the status of the Australian fur seal metapopulation and refine the long-term monitoring program. To that end, we counted annual pup numbers at Seal Rocks between 2013 and 2017, a large colony that had experienced a -28% difference between the 2007 and 2013 census and performed a species-wide census of pup abundance in 2017. We discuss the status of Australian fur seals, provide insights into drivers of metapopulation and local change in pup numbers, and document a case study of an adaptive monitoring program based on a sentinel species, to aid marine conservation.

## Materials and methods

### Species-wide census

Fur seal population estimates are typically based on the number of live pups at breeding sites immediately following the pupping period [45]. This age-class is selected because young pups are the only age cohort available onshore at one time and are easy to handle and distinguish from older seals due to their smaller size, behaviour, and dark natal pelage. Survey design needs to account for site specific variation in population dynamics as well as the compatibility of survey methods across space and time [44, 46]. Therefore, standardised methods at sites are preferred over time to reduce variability between observations.

Australian fur seal population censuses were conducted using three survey methods suitable for the topography of each site: direct ground counts employed at smaller colonies (<1000 pups), capture-mark-resight (CMR) at larger colonies (>1000 pups), and aerial surveys at colonies that were particularly difficult to access on the ground and/or had an open terrain [15, 24] (S2 Table in S1 File). Given the species breeding synchrony, it is reasonable to assume a closed population with equal likelihood of observing all pups at that time given they are of a similar age and stage of development, thus improving the precision of a population estimate and trends [47, 48].

Breeding colonies in Victoria and Tasmania were visited from December 2017 to February 2018 (n = 21, colony descriptions provided in Table 1). The year allocated to data in this paper refers to the year of pup birth (November-December), not the year the survey was performed if occurring in January or February after that birth period. Breeding sites in New South Wales and South Australia are on the edge of the geographical range and were excluded due to logistical constraints; combined, these sites hold 1% of the annual pup production of the species [15]. Note that the name Deen Maar Island is used for Lady Julia Percy Island. In Victoria, the research was performed under animal ethics permit 2.2016 from the Phillip Island Nature Park Animal Ethics Committee and wildlife permits 10007974 and 10003856 from the Department of Environment Land, Water and Planning. In Tasmania, the research was permitted by Department of Natural Resources and Environment through Standard Operating Procedures and management permits for staff.

Capture-Mark-Resight, aerial and direct count techniques have been well developed and applied during the past three censuses in 2002, 2007 and 2013 [15, 24, 37]. The CMR technique uses a modified Petersen formula [49] of marked and clear (unmarked) pups. Marking involved teams of 10–14 people hand-catching pups to clip a triangle of black natal-fur on top of the pup’s head (~32 cm^2^ area). The exposed pale under-fur provides an easily distinguished temporary mark. Effort was distributed evenly across breeding areas, aiming to mark >25% and <50% of pups present for reliable results. Resights of marked and clear pups were performed a minimum of 16 h after marking, to ensure thorough mixing of marked and clear pups, and involved a caller and a scribe moving through the colony or using a vantage point to count the marked and clear pups observed (S2 File). Pups that could not be counted as marked or clear were excluded. Dead pups were recorded as marked or clear during resights and marked dead pups were removed from the total number of marked pups counted for analysis. Natural boundaries of breeding areas were used to separate sub-areas to prevent double counting of mobile pups.

The estimated numbers of pups for sub-areas (*N*) and associated means, and variances were calculated from:

$$N=\left[\frac{\left(M+1)(n+1\right)}{m+1}\right]-1$$

where *M* is the total number of pups that were marked and are available for resighting, *n* is the number of total pups counted, and *m* is the number of marked pups resighted [48]. The estimated total number of pups at a colony was the sum of the means for each sub-area, and total variance was the sum of the corresponding variances.

For direct counts, between three and six independent counts were performed by at least two researchers (S2 File). Researchers moved slowly around the colony and/or from vantage points counted live pups in pre-determined sub-areas, using a hand counter and binoculars. The average of all observers’ total counts was taken as the total number of live pups. In addition to the 5-yearly species-wide census, annual direct pup counts were obtained from 2012 to 2017 at Seal Rocks (S2 File).

Managers often need an estimate of total seal numbers rather than total pup abundance. This requires accounting for pups that have died between birth and the live pup estimate, then a multiplication factor to derive total population from total pup production. The number of pups that die between birth and the date of the estimate, will vary by year and location, and is difficult to measure accurately because tides and waves often carry carcasses away between visits to the colony. At Seal Rocks pup mortality in the first two months of life was estimated in accessible areas from 1966 to the 1970s and averaged 13–15% [50]. In the absence of site and year-specific data for each colony, and to follow previous methodology, 15% pup mortality was added to the estimated number of live pups to estimate total pup production [24, 37]. Gibbens and Arnould [51] calculated a conversion factor of 4.5 times pup numbers to estimate total population size at Kanowna Island. It was based on the age-structure of females, including the low fecundity rate (0.532) observed in this sub-species, and assumed a similar age structure for males. While conversion factors can vary because of spatial and temporal differences in demography and vital rates, this conversion factor is the best available for estimating total population size of this sub-species and was applied to the estimate of total pup production.

### Temporal trends in pup abundance

For several colonies, pup numbers have been estimated by multiple methods over time. Different pup estimation methods vary in precision and final estimated number. For example, direct counts generally result in lower estimates because they assess the number of pups that are seen, while CMR estimates are higher and more precise because they also account for those that cannot be seen [41, 52]. Despite the higher precision and accuracy of CMR methods, direct counts may remain a preferred method because, depending on site specifications, the estimate can be a consistent comparison with CMR (albeit lower) and therefore a valid index of abundance with reduced disturbance and effort [53].

Reliability in trends increases when consistent methods are used at each site, therefore following methods by McIntosh et al. [15], estimates of live pup abundance, consistent in method per breeding colony were used for site specific trends analysis (S2 Table in S1 File). Trends analyses used the results of the four censuses (2002, 2007, 2013 and 2017) and additional data from opportunistic surveys when available (S2 Table S1 File). Opportunistic surveys were checked to ensure they were comparable with the results from the four censuses by site and method.

With recent declines at Deen Maar Island, Kanowna Island has become the second largest breeding colony for the Australian fur seal and, therefore, an important site for monitoring. Both CMR and direct counts have been performed at this site [37, 43], with CMR results published previously for the 2007 and 2013 censuses [15, 24], but not used in these analyses. The most frequent method published for this site are direct counts and a direct count was performed in 2017 to reduce disturbance and will be the method applied for future estimates. An averaged site-specific conversion factor 1.71 (± 0.04) to approximate CMR results from direct counts has been determined from four separate years when both methods were used (2003–05 [43] and 2007 J. Arnould unpubl. data). To allow a reliable comparison, the counts presented in this paper should not be directly compared to the CMR results reported in McIntosh et al. [15]. Being site specific and repeatable, this conversion factor is therefore applied to the direct count data for Kanowna Island to allow a more ‘true’ estimate of total live pup abundance at this site and to improve the estimate of total population size.

Generalised Linear Models (GLMs) were applied to the pup abundance data (response variable) from 1986 to 2018 (explanatory variable) for 19 sites that had ≥3 data points (S2 Table in S1 File) using the package “MASS” (v7.3–45 [54] in the R statistical environment v3.1.1, R Core Team, 2013). Excluded sites with ≤ 3 data points were new sites (Marengo Reef, The Needles, Wendaar Island, Walker Island, Williams Island, Baudin Rocks) or a site where an occasional single pup is born: for example Cape Gantheaume, a frequently monitored long-nosed fur seal colony in South Australia [55]. All GLMs were fitted with a negative binomial distribution to correct for over-dispersion using the log-link function [56]. The use of a negative binomial distribution also avoided the likelihood of standard errors being biased downward, resulting in spuriously large z-values. The negative binomial GLM is not suitable for a small sample size, therefore dispersion parameters (*Initial θ)* were provided to assess confidence in trends. To add further precautions, P values < 0.10 were considered significant, but the percentage deviance explained (*Dev Exp*) and the dispersion parameter (*Initial θ)* of the GLM are also discussed with respect to model significance. The trends for colonies (*Col*) with *Dev Exp* > 50 and *Initial θ* ≤ 10 are considered most reliable, but all trends are informative. The % change (*β Year*, Table 3) for each trend per colony (response variable) was then plotted against the pup abundance estimated in 2017 (explanatory variable). A linear model was applied with 95% confidence intervals to identify any correlation between the pup abundance and the detected change in pup abundance.

To highlight non-linear patterns in the data, a third-order polynomial model was also applied for five sites where this model was expected to perform better than the GLM (Seal Rocks, The Skerries, Reid Rocks, Judgement Rocks and Moriarty Rocks). P-values from the GLM and third-order polynomial were compared, with P < 0.10 and r^2^ > 0.5 considered a good fit for the polynomial and results provided in the S1 and S2 Files.

To further understand the metapopulation trends from the GLMs, the % change (*β Year*, Table 3) over time was calculated for each survey by site and plotted. Zero pup abundance estimates (S2 Table in S1 File) were replaced by “1” to allow the percent change to be calculated for each time point. To aid interpretation, raw data were categorised by the size of pup abundance in 2017 (large > 1700, medium 600:1699 and small 0:599 pups). Cases where a single survey was not categorised with the remainder of the data for that site were correctly allocated and a linear model with 95% confidence intervals applied to show the overall trend for each pup abundance size category.

Finally, a linear regression was applied to the annual direct pup counts (response variable) at Seal Rocks by year from 2012 to 2017 (explanatory variable) (S2 File) to detect the trend. This trend was then compared to the trends determined via CMR at Seal Rocks (SR, Table 3) and the overall pattern of change in pup abundance from the census.

Trends for the sites with greater than 1500 pups, as well as sites with declining trends and/or significant trends are discussed in detail.

## Results

### Species-wide census

**Fig 3:**
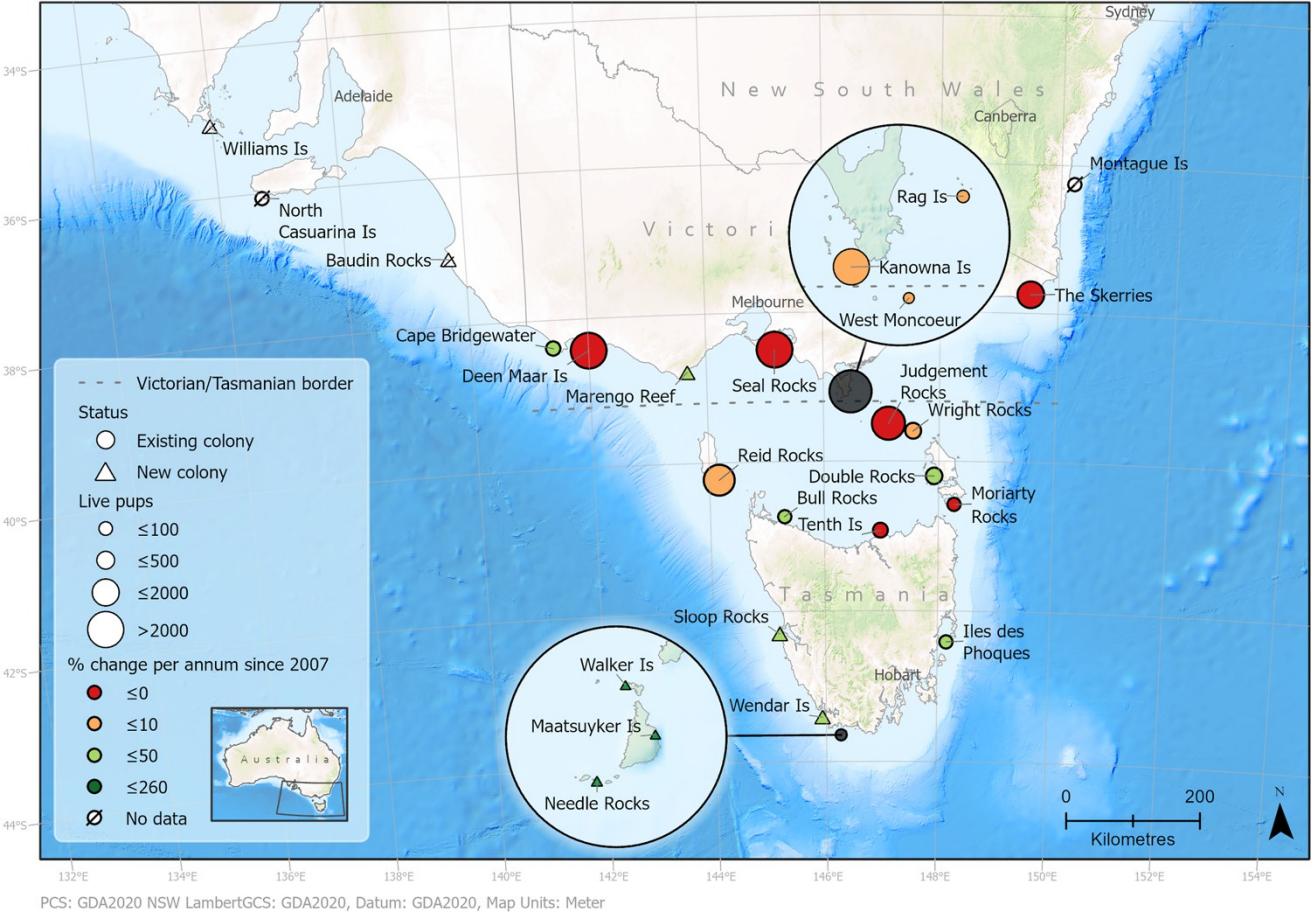
Breeding sites of Australian fur seals indicating the pup abundance and the percentage change in pup abundance per annum between the 2007 and 2017 censuses. The number of live pups is indicated by the size of the shape with larger shapes representing larger pup abundances. The percentage change is indicated by the colour with a scale of red to green indicating negative to positive percent change. Colonies (circles) represent previously identified locations with pups and new colonies (triangles) are those that were identified as having transitioned from a haul-out to a breeding site. Circles of opaque dark grey are extent indicators representing the location of the zoomed detail showing sites in close proximity. The data in this map is from Geoscience Australia [19] and this study.

In 2017, the total number of live pups estimated at 21 sites was 16,903. This compares with 21,589 in 2007 and 17,503 in 2013 (Fig 3 and Table 2). All sites of > 50 pups were monitored in both 2013 and 2017, except for West Moncoeur and North Casuarina in 2017 (which totalled 256 and 75 pups respectively in 2013). The main changes between the 2013 and 2017 censuses were: an inclusion of four new breeding sites in 2017, which had a combined live pup estimate of 372; an increase in pup abundance at Deen Maar Island (8%), Kanowna Island (10%), Cape Bridgewater (41%), Wright Rocks (55%), Tenth Island (74%), and Double Rocks (120%); and fewer pups at Seal Rocks (-5.6%), The Skerries (-29%) and Moriarty Rocks (-83%). Pup abundance in 2017 was below their maximum pup estimate for six sites in 2017: Moriarty Rocks peaked in 1994; Deen Maar Island, Kanowna Island and the Skerries peaked in 2002; Seal Rocks and Tenth Island peaked in 2007. Based on live pup abundance + 15% mortality, 2017 total pup production was 19,836 resulting in a minimum total population for the Australian fur seal in 2017 of 89,300 individuals (rounded to the nearest 100).

**Table 2:** Results of the 2007, 2013 and 2017 Australian fur seal censuses for comparison, grouped by regional oceanographic position or influence (Fig 1) and ordered by maximum negative % change to maximum positive % change between 2013 and 2017, followed by sites without data. The % change of estimated live pup numbers between censuses (2007–2013 and 2013–2017) are presented. Seven recently established sites were identified with pups in 2017 and 2013 and therefore have too few data points for trend analysis. The potential threats were obtained from the references provided at the first mention.

Site (n = 26)	Region	2007 live pups (s.e.)	2013 live pups (s.e.)	2017 live pups (s.e.)	% change between 2007 and 2013	% change between 2013 and 2017	Potential threats
Seal Rocks (SR)	Central Bass Strait	5660 (83)	4092 (38)	3865 (41)	-28	-6	Human waste [22, 57]; sea level rise [23]; entanglement in marine debris [42]; bycatch in trawl fisheries [58]
Kanowna Is. (Kan) *	Central Bass Strait	3078	3382	3239	+10	-4	Foraging overlap with commercial fishing [59]; disease as an abortive agent [60]
Judgement Rocks (JR)	Central Bass Strait	2387 (75)	1710 (24)	1752 (103)	-28	+2	
Rag Is. (Rag)	Central Bass Strait	277^C^	295	351	+7	+19	
Wright Rocks (WR)	Central Bass Strait	130 (01)	187 (02)	289 (7)	+44	+54	
Tenth Is. (TI)	Central Bass Strait	448 (20)	138 (04)	240 (10)	-69	+74	Irregular storm mortality
Double Rocks (DR)	Central Bass Strait	51	157 (02)	346 (3)	+207	+120	
West Moncoeur (WM)	Central Bass Strait	204 (06)	256 (03)	Na	+25	Na	
Deen Maar Is. (DMI)	Bonney Upwelling	5574 (73)	2659 (16)	2866 (24)	-52	+8	Alopecia syndrome [61]; POPs [27]; foraging overlap with commercial fishing
Cape Bridgewater (CB)	Bonney Upwelling	7^C^	120	169	+>1000	+41	Sea cave vulnerable to sea level rise and storm surge
Marengo Reef (MR)	Bonney Upwelling	Na	Na	5	Na	Identified 2017	Irregular storm mortality, sea level rise
North Casuarina (NC)	Kangaroo Island upwelling	28	75 (3.2)	Na	+168	Na	
Cape Gantheaume (CG)^D^	Kangaroo Island upwelling	0	1	0	Incidental observation in 2013	Na	
Williams Is. (WI)	Eyre Peninsula Upwelling	Na	2^C^	Na	Identified 2013	Na	
Reid Rocks (RR)	Western Tasmanian Shelf Upwelling	395^B^	1570 (60)	1568 (9)	+297	0	Irregular storm mortality; overlap with trawl fisheries
Sloop Rocks (Sloop)	Western Tasmanian Shelf Upwelling	0	16	31	Identified in 2013	+94	overlap with trawl fisheries; aquaculture interactions [62]
Bull Rocks (BR)	Western Tasmanian Shelf Upwelling	7	21	44 (1.0)	+200	+109	
Baudin Rocks (Bau)	Western Tasmanian Shelf Upwelling	Na	6^C^	Na	Identified 2013	Na	
Illes des Phoques (IdP)	Sub-Antarctic surface waters	0	10^C^	31 (0)	Na	+210	Aquaculture interactions
Needles (Nde)	Sub-Antarctic surface waters	Na	0	155	Na	Identified 2017	Aquaculture interactions
Walker Is. (Wal)	Sub-Antarctic surface waters	Na	0	96	Na	Identified 2017	Aquaculture interactions
Wendar Is.(Wen)	Sub-Antarctic surface waters	Na	0	45	Na	Identified 2017	Aquaculture interactions
Maatsuyker Is. (Maat)	Sub-Antarctic surface waters	1^C^	0	76	Na	Rapid recent growth	Overlap with trawl fisheries; aquaculture interactions
Moriarty Rocks (MR)	East Australian Current	598 (09)	486 (09)	82 (9)	-19	-83	Irregular storm mortality [63]; strengthening EAC [5, 64]
The Skerries (Ske)	East Australian Current	2705 (31)	2254 (33)^A^	1611 (27)	-17	-28	Irregular storm mortality; foraging overlap with commercial fishing [65]; strengthening EAC; sea level rise [23]
Montague Is. (Mon)	East Australian Current	2^C^	19 (0.3)	Na	+850	Na	Northern-most extent of breeding range [15]
TOTAL SITES SURVEYED		20	26	21			
TOTAL BREEDING SITES		17	21	21			
TOTAL PUPS		21,552	17,456	16,861	-19	-3	

* Direct counts ± s.e. (2017 = 1894 ± 8.24, 2013 = 1978 ± 4.24, 2007 = 1800 ± 6.96) have been converted to CMR using a conversion factor 1.71 to allow more ‘true’ total live pup estimates (2003–5 [43] and 2007 J Arnould unpubl. data).

^A^ Data obtained in 2014 breeding season

^B^ Count differs from Kirkwood et al. [24]. Count confirmed by S. Thalmann, NRE, no s.e. available

^C^ Single direct count

^D^ Incidental observations, one pup also seen in 2012–13, and one hybrid identified in 1995 [66, 67]

### Temporal trends in pup abundance

**Fig 4:**
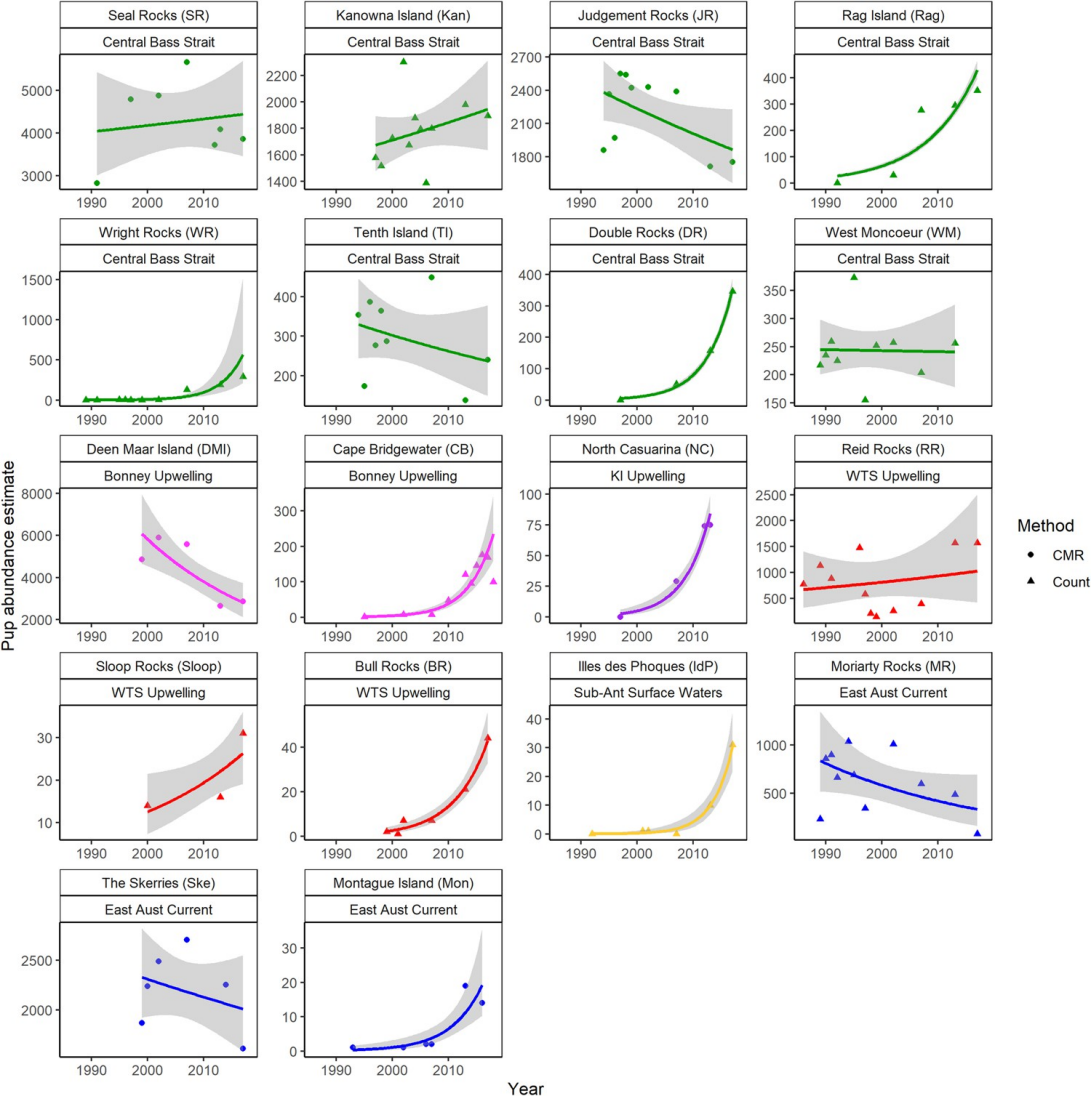
**Smoothed predicted curves (solid line) fitted to estimates of live pup abundance of Australian fur seal pups at breeding colonies in south-eastern Australia, estimated using Generalised Linear Models with negative binomial distributions.** Colony names with abbreviations in brackets and their associated region (also varying by colour) are identified in the heading of each trend and 95% confidence intervals are shown by the grey shading. Live pup abundance estimates used at each site were determined by a single method: direct count or capture-mark-resight (triangle and circle symbols respectively). The sites are grouped by region.

The GLM analyses identified 13 colonies with significant changes in live pup numbers between 2007 and 2017 (Fig 4 and Table 3), although the reliability of these trends for nine of the colonies is questionable due to the few data points available and the highly inflated dispersion parameters (*Initial θ*). Three sites with significant trends from the GLM were reliable: Deen Maar Island (in decline), Rag Island (exponential growth) and Judgement Rocks (in decline) (Fig 4 and Table 3). The GLM model for Maatsuyker Island failed to converge because of few data points (half of which were zeros) and therefore was not included in Table 3 and Fig 4: it has been a haul-out between 1989 and 2013 and experienced rapid growth to 76 pups in 2017.

**Table 3:** Trends analyses from the Australian fur seal live pup abundance estimates including the 2017 census results using Generalised Linear Models. Negative Binomial distribution was applied to live pup abundance data presented in the S2 Table in S1 File; regression results are provided including the percentage deviance explained (*Dev Exp*) and the dispersion parameter (*Initial θ)* of the GLM. The colonies (*Col*) with a significant result at the 0.10 level (shown in bold type), with a Dev Exp >50 and/or Initial *θ* ≤10 represent reliable results and are shaded. Sites are ordered to match Table 2 and Fig 4.

	Negative Binomial GLM							
*Col*	*df*	*β Year*	*Intercept*	*z*	*P*	*- CI*	*+ CI*	*Dev Exp*	*Initial θ*
Seal Rocks	6	0.00	1.15	0.41	0.69	-0.02	0.02	1.9	24.2
Kanowna Is.	10	0.01	-7.60	1.15	0.25	-0.01	0.02	10.7	67.1
Judgment Rocks	9	-0.01	29.00	-2.00	**0.05**	-0.02	0.00	26.6	63.7
Rag Is	4	0.11	-217.74	20.22	**0.00**	0.10	0.12	72.7	1200253.7
Wright Rocks	9	0.25	-500.52	7.80	**0.00**	0.19	0.33	87.9	2.0
Tenth Is.	8	-0.01	34.29	-1.03	0.30	-0.04	0.01	9.4	9.5
Double Rocks	3	0.21	-420.79	16.95	**0.00**	0.19	0.24	97.9	536596.3
West Moncoeur	9	-0.00	6.94	-0.08	0.94	-0.02	0.02	0.1	24.8
Deen Maar Is.	4	-0.04	93.87	-3.40	**0.00**	-0.07	-0.02	67.6	28.8
Cape Bridgewater	9	0.23	-452.30	8.23	**0.00**	0.17	0.29	87.6	6.6
North Casuarina	3	0.22	-431.97	7.58	**0.00**	0.16	0.28	93.5	81458.3
Reid Rocks	10	0.01	-20.79	0.60	0.55	-0.02	0.05	4.1	2.0
Sloop Rocks	2	0.04	-84.45	2.16	**0.03**	0.01	0.09	64.9	3572.5
Bull Rocks	5	0.17	-330.93	7.90	**0.00**	0.13	0.21	94.8	154751.3
Illes des Phoques	5	0.28	-562.33	5.83	**0.00**	0.20	0.39	94.3	49309.4
Moriarty Rocks	10	-0.03	71.44	-1.80	**0.07**	-0.07	0.01	16.8	3.3
The Skerries	5	-0.01	23.98	-0.85	0.40	-0.03	0.01	9.7	38.7
Montague Is.	5	0.18	-361.16	4.00	**0.00**	0.10	0.28	82.7	10.0

For Seal Rocks, The Skerries and Judgement Rocks, the third-order polynomial improved the fit of the model. At all three sites, a period of positive growth and recovery was followed by a recent period of declining pup abundance (S2 Fig and S3 Table in S1 File), a pattern clearly present in the data points of Fig 4 for these sites.

Regional influences are not clearly affecting trends across sites, with all regions except the Western Tasmanian Shelf Upwelling (WTSU) showing varied trends (Table 2, Fig 4). Some sites in southern Tasmania, with the regional influence of the sub-Antarctic surface waters (SASW), have too few data points for trends analysis, except for Illes des Phoques which is a small rapidly increasing colony (Fig 4).

**Fig 5:**
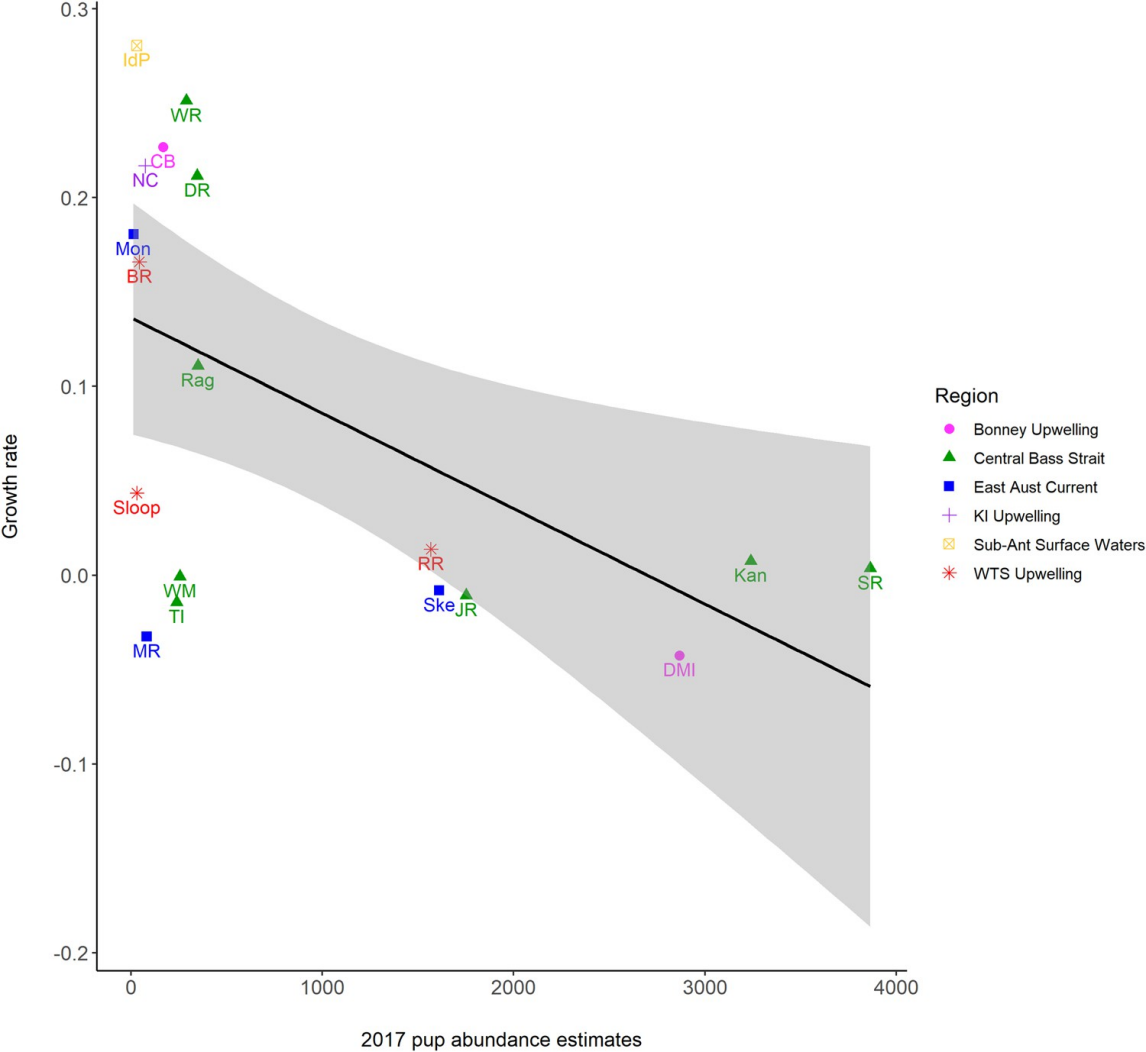
**Scatterplot and linear regression (black line) of the rate of change (Growth rate or *β Year* Table 3) of each breeding colony (shown using abbreviations defined in Table 1 and Fig 4) in response to the 2017 pup abundance estimate, with the 95% confidence interval shown by grey shading.** Symbols and colours of sites represent the different oceanographic regions where they are situated.

A significant negative relationship (r^2^ = 0.308, F = 7.121 _1,16_, p = 0.017) was identified between the rate of change (*β Year*, Table 3) for each GLM trend per colony and the pup abundance estimated in 2017, as the most recent pup abundance estimate (Fig 5). This identifies that the sites with larger pup abundance had smaller but more negative rates of change than the more recently colonised sites, many of which were rapidly increasing. There is no influence of region on the rate of change by site (Fig 5).

To further explore this relationship, the % change between surveys for each site were categorised by the size of pup abundance in 2017 (large > 1700, medium 600:1699 and small 0:599 pups) and a linear smoother applied to the % change between surveys over time. Indeed, larger sites did show a significant negative trend over time (r^2^ = 0.199, F = 7.949 _1, 32_, p = 0.008) and medium and small sites showing non-significant neutral and slight positive trends over time (r^2^ < 0.000 and r^2^ = 0.016 respectively) (S1 Fig in S1 File).

The trend in annual pup counts at Seal Rocks between 2012 and 2017 may present a slight decline or be stable, with overlapping error bars of averaged counts. The linear regression shows some variation (r^2^ = 0.392, y = -124.46x + 253627) with an average of 2908 ± 372 (SD) live pups counted (S2 Fig and S3 Table in S1 File). Although there are only three paired datapoints with CMR surveys (2012, 2013 and 2017) the correlation is high (r^2^ = 0.765) indicating that direct counts generally reflect CMR surveys with an average conversion factor of 0.69 (0.70, 0.71 & 0.66 respectively). The counts show low variability and therefore support the hypothesis that pup numbers have remained at a lower level than in 2007, over the six years from 2012 and the reduction first recorded at Seal Rocks in 2012 has been sustained.

## Discussion

Since the extirpation of the seals and their protection in 1975, Australian fur seal pup numbers recovered to a peak of around 21,552 in 2007, possibly one half of the estimated original population size (Fig 2). The 2013 and 2017 censuses, as well as annual pup counts at Seal Rocks, identify that pup abundance has reduced overall and may be stabilising at around 16,900 to 17,500 live pups. Although a generalised estimate, the minimum total population size in 2017, was 89,500 fur seals, representing a 25% reduction from the 2007 population estimate of 120,000 individuals. Despite the overall reduced pup abundance, the number of known breeding sites has increased over the three censuses from 19 to 21 and is now known to be 26 breeding sites, representing an expansion of breeding range, largely in Tasmania.

### Observed trends in pup abundance

Overall, there are four main types of trends occurring at breeding sites: increasing pup abundance at the recently colonised sites, reduced pup abundance in the large and well-established sites (except for Kanowna Island); neutral sites without change and variable pup abundance at the sites with stochastic population changes where pup abundance fluctuates with natural events.

Sites with significant and reliable positive trends are Rag Island, Wright Rocks, Double Rocks, Cape Bridgewater, Bull Rocks, Illes des Phoques and Montague Island (Table 3). These sites occur across multiple regions: Central Bass Strait, Bonney Upwelling, Western Tasmanian Shelf Upwelling, sub-Antarctic surface waters and the East Australian Current. However, all produce fewer than 500 pups and are considered small colonies or establishing sites.

The sites with significant and reliable negative trends are Deen Maar Island in the Bonney Upwelling region and Moriarty Rocks in the region of the East Australian Current. Deen Maar Island has been the site of highest pup abundance (5574 pups in 2007) across the range until 2013 when a 52% reduction was detected. Moriarty Rocks has supported over 1000 pups prior to the early 2000s, but pup numbers have reduced since and an 83% reduction was detected between 2013 and 2017. These trends are concerning.

It was necessary to use GLMs for all sites to allow a full comparison of rate of change; however, for Seal Rocks, The Skerries and Judgment Rocks, a better model fit was the cubic polynomial because the trends at these sites entered a period of decline after a period of growth. All three sites have high pup production and are therefore important for the species. These sites typically increased in the 1990’s, with growth slowing before peaking around the 2000’s and then reducing by 2012–2013, remaining around the new reduced level in 2017. While the reduced pup abundance for the total population and Seal Rocks was detected after 2007, reduced pup abundance may have commenced earlier at Deen Maar Island and Judgment Rocks and later at The Skerries. The annual pup counts at Seal Rocks from 2012–2017 confirm that the reduction in pup numbers observed during the 5-yearly censuses represent a true drop in pup abundance and not simply the monitoring of poor seasons by happenchance.

Kanowna Island, with the second largest pup abundance and relatively close to Seal Rocks, is the only large breeding site that has not shown a decreasing trend in pup numbers (Table 3 and Fig 4). There is some site fidelity in foraging range for this species [65], therefore local conditions within regions may be influencing the variable trends observed at the larger breeding colonies.

Sites such as Reid Rocks, Moriarty Rocks and Tenth Island (Table 1) are low-lying and pup numbers are known to be negatively impacted by storm-surges in some years [68]. The stochasticity of such sites is less useful for understanding drivers of population change. Change in pup abundance may be caused by change in birth and/or survival rates and/or a redistribution of breeding females. It is also likely that threats specific to certain sites are affecting pup numbers and some drivers are synergistic (Table 2).

### Drivers of metapopulation trends for the Australian fur seal

Several sites were not surveyed in the 2017 census, but these sites do not have high numbers of pups and therefore would not have influenced the overall reduction in pup abundance. These sites included West Moncoeur in Tasmania (256 pups in 2013) and North Casuarina in South Australia (75 pups in 2013), as well as the recently established sites in South Australia: Baudin Rocks (6 pups in 2013) and Williams Island (2 pups in 2013: perhaps a site with only occasional pupping), and New South Wales at Montague Island (14 pups in 2016) (S2 Table in S1 File). It is also possible that some emerging colonies, particularly in Tasmania have not yet been identified. This total of 353 pups does not account for the reduction in pup numbers by 4,691 pups between 2007 and 2017 pup abundance estimates (Table 2). This also explains why it is unlikely to be a redistribution of the metapopulation. Such a redistribution would likely occur in response to reduced access to resources such as suitable breeding sites and/or food resulting in migration to sites with greater resource availability. However, since monitoring began, we have not detected carrying capacity of the metapopulation, for example by a sustained maximum population or the asymptote of a logistic curve [69, 70] (Fig 2). Instead, in 2007, it was thought that the population growth may be slowing down, and in 2013 a reduction in overall pup abundance was reported [15, 24]; an indicator that may be cause for concern regarding the health of the metapopulation [40].

Should a population reach breeding capacity for limited habitat or deplete their local food resources, an ‘overshoot’ or negative breeding response may be observed, as has been reported for a population of northern fur seals (*Callorhinus ursinus*) [71]. High density in breeding areas can force females into sub-optimum breeding habitat, resulting in reduced pup body condition, or encouraging dispersal to an alternative site [72, 73]. For Australian fur seals, it is unlikely that the population has reduced in response to overcrowding, or localised resource depletion. Using records from commercial harvesting of Australian fur seals, the population was over twice as large as it is now, roughly estimated at over 200,000 individuals [74, 75] (Fig 2). Additionally, the Australian fur seal is closely related to the Cape fur seal, that lives in colonies of high density (0.92 pups per m^2^) compared to the Australian fur seal (0.59 pups per m^2^) [23, 76, 77]; presumably, the Australian fur seal is capable of similar behaviour, provided access to sufficient food resources.

As a generalisation and excluding the small colonies at edges of the breeding range and Kanowna Island, the sites with declining and or reduced pup abundance are situated north of the Tasmanian land mass (42.5°S). It is plausible that food resources are affecting Australian fur seal pup abundance at these sites. Australian fur seals are broadly understood to be an opportunistic predator with diet plasticity. Supporting this, the diet at Seal Rocks has changed since 1997 in response to large-scale climate and oceanographic processes [25, 78, 79]. Climate change is predicted to affect ocean temperature, currents and upwelling periods that will alter food webs [80] and eastern Bass Strait is a hotspot of global warming [5]. In additional to oceanographic change, industrialised fishing in eastern Australia has been linked to reduced biodiversity and biomass of species in the region [81, 82].

Both local- and large-scale environmental variability influences foraging and therefore the reproductive success of female Australian fur seals, with both predicted to become more difficult for females in the near future [26]. In addition to this, increased storm surges and rising sea levels as a result of climate change are predicted to inundate the breeding habitat at many colonies further impacting pup survival, including two of the largest: Seal Rocks and The Skerries where pup numbers have already reduced [23]. Fur seals in New South Wales, Bass Strait, and South Australia may need to shift to sites with higher ground and/or move further south to physiologically cope with increased storm surge, rising sea levels, summer heat waves and climate induced prey shifts [26, 83]. Strong population growth at recently colonised sites is expected for fur seals exploiting new sites, assuming available food and breeding habitat [71, 84]. With the rapid increase in pups observed in southern Tasmania, this may already be occurring.

Compounding these threats, the Australian fur seal has the lowest fecundity observed in fur seals, with a mid-gestation pregnancy rate of 84% (based on progesterone concentrations) and a high abortion rate resulting in a low birth rate (53%) [85, 86]. Also, other threatening processes are emerging. For example, persistent organic pollutants (POPs) some of which have both endocrine and reproductive effects have been detected in juvenile and adult female Australian fur seals at Deen Maar Island and Seal Rocks (both sites with reduced pup numbers) with survival rates being impacted [22, 27]. More recently, research has identified PFAS at high concentrations in pups sampled at Seal Rocks [22]. The adverse health impacts of PFAS are yet to be elucidated but they may affect breeding rates or pup survival through disease and/or endocrine disruption [27, 61]. Also, human-associated antibiotic resistant bacteria have been found at very high levels at Seal Rocks, indicating that exposure to human waste is influential for this site [87].

Further anthropogenic threats include entanglement in marine debris and mortality in fishing bycatch. Marine debris entanglement incidence is high and predominantly affects juveniles and pups, which can reduce recruitment and pup production [42, 88]. In addition, an estimated 700 fur seals per year (most likely to be Australian fur seals, based on their range overlap) are incidentally caught by vessels of the South East Trawl Fishing Industry Association [58, 89, 90]. It is possible to mitigate fishing interactions through gear changes and/or spatial closures around breeding colonies as demonstrated in South Australia with the Australian sea lions [91], although it comes at a cost to the fishing industry and every fishery has unique characteristics that may or may not make such mitigation feasible.

The current trends include the establishment of new colonies on the edges of the range and likely some redistribution of individuals between 2013 and 2017. Three new breeding sites were identified in South Australia and Victoria since 2007; however, within southwest Tasmania, the rapid establishment of breeding sites and their high rate of increase is unprecedented (Fig 3; S2 Table in S1 File). The Needles, Maatsuyker, Wendar and Walker Islands have transitioned from being haul-out locations to breeding colonies within a two-year period and in 2017 accounted for 372 pups, with strong growth continuing in subsequent years (S. Thalmann, Department of Natural Resources and Environment Tasmania—NRE, unpubl. data). Consequently, pup abundance in Tasmania now accounts for 28% of the total live pup abundance compared to 19% in 2007. The scale of the overall reduction in pup abundance at the large colonies of Bass Strait since 2007 cannot be solely explained by redistribution of breeding females and it would be misleading to consider the increase in Tasmania in isolation. Within Tasmania there are significant management complexities involving fur seal populations and interactions with aquaculture [62, 92], and these are likely to increase in line with potential future growth of both industry and fur seal populations in this region.

### Implications for future monitoring

Future monitoring would ideally target sites where change in pup abundance is indicating broad ecosystem change that is poorly understood. These are the large colonies with individual trends, plus recently established and increasing populations. Key sites in each region include:

1. Western Victoria near the Bonney Upwelling Zone–where pup production at Deen Maar Island has halved and an alopecia syndrome linked to environmental toxicity is present [15, 27, 61].
2. Central Victoria in Bass Strait–where pup abundance at Seal Rocks has reduced by one third, but pup abundance at the adjacent Kanowna Island remains stable or increasing; these colonies are now of similar size, when historically, Seal Rocks has been significantly larger [24].
3. East coast of Victoria in the strengthening East Australian Current and a hotspot of ocean warming [5, 64]–where a further 28% reduction in pup abundance was detected at The Skerries in 2017.
4. Maatsuyker group, Tasman Fracture Marine Park and the Sub-Antarctic surface waters–the area of current population growth that is adjacent to fish farming activities [62].

A 5-yearly census of all the sites and more frequent assessments (ideally annual) of the above prioritized sites, should be a minimum program requirement; acknowledging however, that programs may be adjusted depending on other jurisdictional decisions and resourcing. Breeding sites with low pup abundance at the edges of the range in South Australia and New South Wales will continue to be included in the census when possible and otherwise monitored opportunistically. It is important to recognise that selecting key sites to represent an aggregate of sites can bias the results because of spatial heterogeneity in vital rates such as pup survival [93]; however, a complete 5-yearly census should account for this. In the case of the adaptive monitoring program for the Australian fur seal, the 5-yearly census of all sites across the range ensures an understanding of the broader metapopulation health and trajectory and allows us to continue to adapt the monitoring program in the event of further change.

It is anticipated that Australian fur seal populations will respond to warming climate by migrating southwards. In Tasmania, Judgment Rocks and Wright Rocks in Central Bass Strait were previously identified as priority sites for population monitoring due to the larger population size and the opposing trends being expressed [15]. However, the southern Tasmanian aquaculture industry continues to grow in production volume and spatial distribution [94] and new colonies may be emerging, in part, in response to this expansion with associated management challenges [62]. Therefore, a suite of larger established colonies and emerging colonies adjacent to aquaculture production zones (Ile Des Phoques, Maatsuyker, Needles, Wendar, Walker Islands and Sloop Rocks: Fig 3) should be prioritized for monitoring in Tasmania. Furthermore, within central Tasmanian Bass Strait, Tenth Island is a colony of high stochasticity [68] making it a poor candidate for trend analysis. However, pup body condition indices have been collected there since 2003 and continued monitoring would provide valuable comparisons with other sites (S. Thalmann NRE pers. comm).

The Bass Strait ecosystem has been affected by industrialised fishing, reducing species biomass (amount of living tissue) and diversity (number of species) [81, 82]. The Australian fur seal is an apparently abundant predator in south-east Australia—but its population is estimated at half of the estimated population 200 years ago (Fig 2) [75]. As a community and as managers, we are suffering from the shifting baselines syndrome where we consider the current population to be high because we are comparing current seal numbers to those present 40 years ago instead of those present over 200 years ago. Not isolated to the fur seals, this syndrome results in flawed measures of sustainable resource extraction (e.g. fish stocks) and inadequate protection of species [9, 95]. As an obvious example, many commercial fisheries developed during the period of low seal numbers now view seals as over-abundant competitors [81, 96, 97].

Marine conservation has the difficult challenge of mitigating anthropogenic impacts in a time of rapid change. It is critical to be inclusive and responsive while generating reliable data for decision making. Ethical future research should include collaboration and engagement with Traditional Custodians whose responsibilities in caring for Country and animals such as the fur seals are significant as outlined in Sea Country and Country Plans [98, 99]. The unique knowledge systems of Aboriginal and Torres Strait Islander peoples and can only improve research and management of marine ecosystems and there are guidelines available from the Australian Institute of Aboriginal and Torres Strait Islander Studies to develop opportunities, a process endorsed by the Australian Marine Science Association [100–102]. Engagement between industry, scientists, managers, and First Nations people, will be critical to rethink ecosystem function, adaptively respond to change, and offset the negative influence of shifting baseline syndrome.

## Conclusions

Pup abundance for the recovering Australian fur seal metapopulation appears to have reached a peak around 2007 and is now entering a period of change that may include a southward range shift and overall reduced pup production. The observed decline in pup abundance may be attributed to reduced reproductive success, reduced recruitment and/or increased adult mortality. Contributing factors likely include: the warming climate with associated heat waves, sea level rise and disruption of food webs; altered ecosystems through fishing; fisheries bycatch mortality; entanglement in marine debris and disease impacts including ecotoxicity. Demographic research is currently underway to better understand the drivers of change. The observed reduction in pup numbers will translate into reduced future recruitment to the breeding population and further population reductions in southern Australia that could have flow-on consequences for the stability of the ecosystem.

This paper highlights the importance of critically examining monitoring programs with the aim of adapting and optimising them at regular intervals. In doing so, the potential for monitoring programs to achieve their goals and provide reliable data and predictions for conservation and management is enhanced.

## Supporting information

S1 File Supporting information S1-S3 Tables and S1-S3 Figs. (DOCX)

S2 File Supporting information raw data. (XLSX)
