# Supplementary material for: Sustained reduction in numbers of Australian fur seal pups: Implications for future population monitoring
Source: PLoS One. 2022 Mar 18;17(3):e0265610. doi: 10.1371/journal.pone.0265610 (PMC8932563; doi:10.1371/journal.pone.0265610)
Supplement: S1 File — (DOCX) [file pone.0265610.s001.docx]

**S1 File**

**S1 Table. Estimated Australian fur seal (*Arctocephalus pusillus doriferus*) pup numbers, upper and lower estimate of pup numbers and confidence intervals (CI) when determined from 1750 to 2013, as well as the references used to approximate the numbers.** Data from 1750-1991 are not accurate or precise, but largely assumed based on limited recorded information until 1945 when the first census of the population was performed. Confidence intervals were unknown until the 1986 census, it was expected that any pup abundance estimate was more likely to be a minimum than a maximum, therefore unknown CIs were calculated to be up to 50% higher or 20% lower than the associated estimate. Where pup estimates are based on census survey data (1991-2013), standard errors of the pup estimate are used to determine 95% CIs.

| **Year** | **Australian fur seal pup estimate** | **Upper pup estimate** | **Lower pup estimate** | **+95% CI** | **-95% CI** | **Source** | **Comment** |
| --- | --- | --- | --- | --- | --- | --- | --- |
| 1750 | unknown |  |  |  |  | Stockton 1982 | Hunting and cultural use by First Nation Australians for ~8,000 years |
| 1750 | 37,000 | 52000 | 20000 |  |  | Warneke 1982, Ling 2002, Shaughnessy and Warneke 1987 | Total fur seal population estimated from ship records of skins for Bass Strait and King Island and rounded to the nearest 50 = 243,900 seals to 350,000 seals. Estimated number of pups calculated by dividing by 4.5 (pup multiplier) and assuming two thirds were Australian fur seals. This is expected to be a minimum because many skins were unaccounted, and seals killed without record. |
| 1798 | 37,000 | 52000 | 20000 |  |  | Warneke 1982, Lewis 1929 | 1798 onset of Commercial industry with the boom passing by 1810 |
| 1830 | 1500 | 2250 | 1200 |  |  | Ling 2002 | 96% of the total harvest of fur seal taken by 1820 |
| 1840 | 1500 | 2250 | 1200 |  |  | Warneke 1982, Ling 2002, Shaughnessy and Warneke 1987 | Subsistence industry for commercial sealing |
| 1868 | 500 | 750 | 400 |  |  | Warneke 1966 | 100 individuals present on Seal Rocks in 1860 |
| 1889 | 500 | 750 | 400 |  |  | Shaughnessy and Warneke 1987 | Government control of seal harvesting under the Fisheries Act of 1889 in Tasmania and the Game Act of 1890 in Victoria |
| 1903 | 2000 | 3000 | 1600 |  |  | Lewis 1929 | 20 seals on Seal Rocks, accuracy unknown |
| 1925 | 3000 | 4500 | 2400 |  |  | LeSouef 1925, Arnould et al. 2003, Shaughnessy and Warneke 1987 | 13,215 total Australian fur seals counted. Commercial harvests ceased by 1920 but seals culled for interfering with commercial fishing and from 1923-1983 fishers not penalised for killing “nuisance seals”. |
| 1928 | 5000 | 7500 | 4000 |  |  | Lewis 1929 | 5000-6000 seals, 600 pups at Seal Rocks & 3000-5000 seals, 1000 pups at Lady Julia Percy Island = 1600 pups, Seal Rocks pup number seems too low for total seals and assumed other sites have pups as well. |
| 1945 | 10000 | 15000 | 8000 |  |  | Pearse 1975, Shaughnessy and Warneke 1987, Warneke 1988 | First census performed by aerial survey but outside the breeding season so known to be an underestimate. Pearse 1975 aerial count adjusted by 1.8 to compare to a ground survey during the breeding season. |
| 1962 | 7000 | 10500 | 5600 |  |  | Arnould et al. 2003 | A dip in survival rates and population size predicted between 1954 and 1971, particularly in the mid-early 1960’s. Population unknown. |
| 1975 | 10000 | 15000 | 8000 |  |  | Pearse 1975, Warneke 1979, Warneke and Shaughnessy 1985, Shaughnessy and Warneke 1987 | Seals protected under the 1957 Wildlife Regulations of the National Parks and Wildlife Act of 1970. From 1966-1985, number of seal pups at Seal Rocks stable at 2000 |
| 1986 | 10000 | 15000 | 8000 |  |  | Warneke 1988, Shaughnessy and Warneke 1987 | Aerial survey expected to be an underestimate and adjusted to compare to ground count |
| 1991 | 13335 |  |  | 13696 | 12974 | Pemberton and Kirkwood 1994 | Total pup number estimated from Tasmanian census, 95% CI calculated from the standard error of the pup estimate in 2002 (SE = 184) revised by Kirkwood et al. (2010). |
| 1995 | 16893 |  |  | 17254 | 16532 | Kirkwood et al. 2005 | Estimates based on counts in different years, 95% CI calculated from the standard error of the pup estimate in 2002 (SE = 184) revised by Kirkwood et al. (2010). |
| 1998 | 17126 |  |  | 17487 | 16765 | Kirkwood et al. 2005 | Estimates based on counts in different years, 95% CI calculated from the standard error of the pup estimate in 2002 (SE = 184) revised by Kirkwood et al. (2010). |
| 2002 | 21545 |  |  | 21906 | 21184 | Kirkwood et al. 2005 | Full census using ground counts, capture-mark-resight and aerial surveys, 95% CI calculated from the standard error of the pup estimate in 2002 (SE = 184) revised by Kirkwood et al. (2010). |
| 2007 | 21882 |  |  | 22249 | 21515 | Kirkwood et al. 2010 | Full census using ground counts, capture-mark-resight and aerial surveys, 95% CI calculated from the standard error of the pup estimate in 2002 (SE = 187). |
| 2013 | 16503 |  |  | 16803 | 16203 | McIntosh et al. 2018 | Total pup estimate adds the 2014 estimates from The Skerries and Maatsuyker Island that were missed in the 2013 census. |

**S2 Table. Annual time-series data of Australian fur seal live pup abundance per colony that were available for this study including direct ground counts (C), aerial surveys (Aer) and capture-mark-resight estimates (CMR).** Data for each site represent the single method (Meth) most used and only include surveys that employed that method at that site. Being a large colony, the direct counts at Kanowna Island (Kan, n=10) have been multiplied by the site-specific multiplier of 1.71 to approximate a CMR and provide more ‘true’ estimates of total pup numbers for the sub-species. The total pups are only provided for the four censuses (2002, 2007, 2013 and 2017). Note: the 2013 total does not include the large colony The Skerries, which was sampled the following year. Site name abbreviations are identified in the postscript. Data were cross-checked, some were obtained from individual agencies and published records were used. Year is from the start of that breeding season (November).

| **Site** | **DMI** | **SR** | **Kan** | **Ske** | **Rag** | **CB** | **Mar** | **RR** | **WM** | **JR** | **TI** | **MR** | **WR** | **DR** | **BR** | **IdP** | **Sloop** | **Maat** | **Wen** | **Nde** | **Wal** | **NC** | **WI** | **Bau** | **CG** | **Mon** | **Total** |
| --- | --- | --- | --- | --- | --- | --- | --- | --- | --- | --- | --- | --- | --- | --- | --- | --- | --- | --- | --- | --- | --- | --- | --- | --- | --- | --- | --- |
| **Meth** | CMR | CMR | C  *1.71 | CMR | C | C | C | Aer | C | CMR | CMR | C | C | C | C | C | C | C | C | C | C | CMR | C | C | C | CMR |  |
| **1986** |  |  |  |  |  |  |  | 775 |  |  |  |  |  |  |  |  |  |  |  |  |  |  |  |  |  |  |  |
| **1987** |  |  |  |  |  |  |  |  |  |  |  |  |  |  |  |  |  |  |  |  |  |  |  |  |  |  |  |
| **1988** |  |  |  |  |  |  |  |  |  |  |  |  |  |  |  |  |  |  |  |  |  | 0 |  |  |  |  |  |
| **1989** |  |  |  |  |  |  |  | 1131 | 217 |  |  | 234 | 1 |  |  |  |  | 0 |  |  |  | 0 |  |  |  |  |  |
| **1990** |  |  |  |  |  |  |  |  | 235 |  |  | 858 |  |  |  |  |  |  |  |  |  |  |  |  |  |  |  |
| **1991** |  | 2826 |  |  |  |  |  | 885 | 259 |  |  | 897 | 1 |  |  |  |  |  |  |  |  |  |  |  |  |  |  |
| **1992** |  |  |  |  | 0 |  |  |  | 225 |  |  | 665 |  |  |  | 0 |  |  |  |  |  | 0 |  |  |  |  |  |
| **1993** |  |  |  |  |  |  |  |  |  |  |  |  |  |  |  |  |  |  |  |  |  |  |  |  |  | 1 |  |
| **1994** |  |  |  |  |  |  |  |  |  | 1859 | 354 | 1035 |  |  |  |  |  |  |  |  |  | 0 |  |  |  |  |  |
| **1995** |  |  |  |  |  | 1 |  |  | 373 | 2365 | 173 | 689 | 3 |  |  |  |  |  |  |  |  | 0 |  |  |  |  |  |
| **1996** |  |  |  |  |  |  |  | 1476 |  | 1971 | 386 |  | 3 |  |  |  |  |  |  |  |  |  |  |  |  |  |  |
| **1997** |  | 4794 | 2700 |  |  |  |  | 579 | 155 | 2548 | 277 | 345 | 1 | 0 |  |  |  |  |  |  |  | 0 |  |  |  |  |  |
| **1998** |  |  | 2592 |  |  |  |  | 210 |  | 2539 | 364 |  |  |  |  |  |  |  |  |  |  |  |  |  |  |  |  |
| **1999** | 4867 |  |  | 1867 |  |  |  | 142 | 252 | 2421 | 287 |  | 1 |  | 2 |  |  |  |  |  |  |  |  |  |  |  |  |
| **2000** |  |  | 2953 | 2237 |  |  |  |  |  |  |  |  |  |  |  |  |  |  |  |  |  |  |  |  |  |  |  |
| **2001** |  |  |  |  |  |  |  |  |  |  |  |  |  |  | 1 | 1 |  |  |  |  |  |  |  |  |  |  |  |
| **2002** | 5899 | 4882 | 3935 | 2486 | 30 | 7 |  | 259 | 257 | 2427 |  | 1007 | 5 |  | 7 | 1 |  |  |  |  |  |  |  |  |  | 1 | 21203 |
| **Site** | **DMI** | **SR** | **Kan** | **Ske** | **Rag** | **CB** | **Mar** | **RR** | **WM** | **JR** | **TI** | **MR** | **WR** | **DR** | **BR** | **IdP** | **Sloop** | **Maat** | **Wen** | **Nde** | **Wal** | **NC** | **WI** | **Bau** | **CG** | **Mon** |  |
| **2003** |  |  | 2861 |  |  |  |  |  |  |  |  |  |  |  |  |  |  |  |  |  |  |  |  |  |  |  |  |
| **2004** |  |  | 3210 |  |  |  |  |  |  |  |  |  |  |  |  |  |  |  |  |  |  |  |  |  |  |  |  |
| **2005** |  |  | 3068 |  |  |  |  |  |  |  |  |  |  |  |  |  |  |  |  |  |  |  |  |  |  |  |  |
| **2006** |  |  | 2370 |  |  |  |  |  |  |  |  |  |  |  |  |  |  |  |  |  |  | 11 |  |  |  | 2 |  |
| **2007** | 5574 | 5660 | 3078 | 2705 | 277 | 7 |  | 395 | 204 | 2387 | 448 | 598 | 130 | 51 | 7 | 0 | 0 | 1 |  |  |  | 28 |  |  | 0 | 2 | 21552 |
| **2008** |  |  |  |  |  |  |  |  |  |  |  |  |  |  |  |  |  |  |  |  |  |  |  |  |  |  |  |
| **2009** |  |  |  |  |  |  |  |  |  |  |  |  |  |  |  |  |  |  |  |  |  |  |  |  |  |  |  |
| **2010** |  |  |  |  |  | 47 |  |  |  |  |  |  |  |  |  |  |  |  |  |  |  |  |  |  |  |  |  |
| **2011** |  |  |  |  |  |  |  |  |  |  |  |  |  |  |  |  |  |  |  |  |  | 74 |  |  |  |  |  |
| **2012** |  | 3725 |  |  |  |  |  |  |  |  |  |  |  |  |  |  |  |  |  |  |  |  |  |  |  |  |  |
| **2013** | 2659 | 4092 | 3382 |  | 295 | 120 |  | 1570 | 256 | 1710 | 138 | 486 | 187 | 157 | 21 | 10 | 16 | 0 | 0 | 0 | 0 | 75 | 2 | 6 | 1 | 19 | 15202 |
| **2014** |  |  |  | 2254 |  | 95 |  |  |  |  |  |  |  |  |  |  |  |  |  |  |  |  |  |  |  |  |  |
| **2015** |  |  |  |  |  | 146 |  |  |  |  |  |  |  |  |  |  |  |  |  |  |  |  |  |  |  |  |  |
| **2016** |  |  |  |  |  | 176 |  |  |  |  |  |  |  |  |  |  |  |  |  |  |  |  |  |  | 0 | 14 |  |
| **2017** | 2866 | 3865 | 3239 | 1611 | 351 | 169 | 5 | 1568 |  | 1752 | 240 | 82 | 289 | 346 | 44 | 31 | 31 | 76 | 45 | 155 | 96 |  |  |  | 0 |  | 16861 |
| **2018** |  |  |  |  |  | 99 |  |  |  |  |  |  |  |  |  |  |  |  |  |  |  |  |  |  |  |  |  |

Note: the 2000 survey for Kanowna Island was listed as a CMR in McIntosh et al. (2018), this survey was a direct count. Tasmanian data provided by Department of Primary Industries and Parks, Water and Environment (DPIPWE) and Pemberton and Kirkwood (1994), Pemberton and Gales (2004), Kirkwood et al. (2005), Kirkwood et al. (2010). Victorian data from Arnould and Littnan (2000), Littnan and Mitchell (2002), Shaughnessy et al (1995, 2000, 2002), Sorrell et al. (2019), Stamation et al. (1997), Kirkwood et al, 2005, 2010) and McIntosh et al. (2018) and this study. Data for South Australian sites in McIntosh et al. (2018), Goldsworthy et al. (2016 & 2017), Shaughnessy and Goldsworthy (2012), Shaughnessy et al. (2010). Data for NSW is from Shaughnessy et al. (2001) and McIntosh et al. (2018). Raw data provided in S2 File.

Colony names and abbreviations by state of Australia:

**Victoria;** Deen Maar Island (DMI, also named Lady Julia Percy Island), Seal Rocks (SR), Kanowna Island (Kan), The Skerries (Ske), Rag Island (Rag), Cape Bridgewater (CB) and Marengo Reef (Mar).

**Tasmania;** Reid Rocks (RR), West Moncoeur (WM), Judgment Rocks (JR), Tenth Island (TI), Moriarty Rocks (MR), Wright Rocks (WR), Double Rocks (DR), Bull Rock (BR), Sloop Rocks (Sloop), Iles des Phoques, (IdP), Maatsuyker (Maat), Wendaar Is (Wen), Needles (Nde) and Walker Is (Wal).

**South Australia;** Williams Is (WI), North Casuarina (NC), Cape Gantheaume, Baudin Rocks (Bau).

**New South Wales;** Montague Island (Mon).


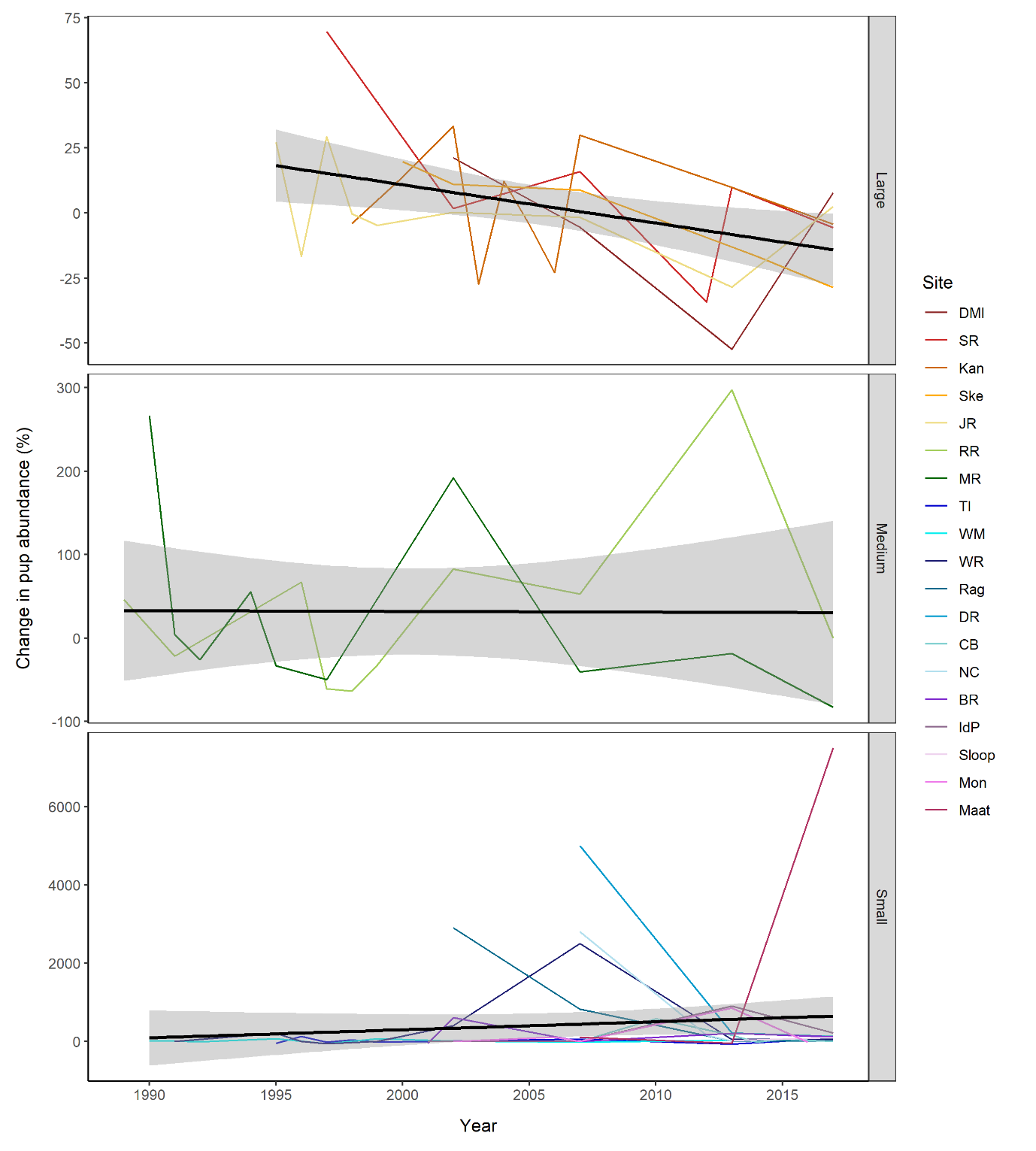


**S1 Fig. Percent change in pup abundance between individual surveys for each breeding site of Australian fur seals from 1986-2017.** Data were categorised by the size of pup abundance in 2017 as large >1700, medium 600:1699 and small 0:599 number of pups; site abbreviations are described in S2 Table. A linear model (black line) with 95% confidence band (grey) is fitted to each size group of pup abundance to show the overall trend in population change (%) for each group.

**S3 Table. Trends analyses from the Australian fur seal live pup estimates including the 2017-18 census results using third-order polynomial linear model.** Models were applied to pup abundance data presented in the S1 Table. Sites with trends at significance of p < 0.10 and r^2^ > 0.5 are shaded.

|  | **Third-order Polynomial Linear Model** | | | |
| --- | --- | --- | --- | --- |
| *Colony* | *df* | *R^2^* | *f-statistic* | *P* |
| Seal Rocks | 4 | 0.58 | 5.23 | 0.077 |
| The Skerries | 3 | 0.95 | 47.51 | 0.005 |
| Reid Rocks | 8 | 0.33 | 3.42 | 0.084 |
| Judgement Rocks | 7 | 0.51 | 5.64 | 0.035 |
| Moriarty Rocks | 8 | 0.26 | 2.77 | 0.122 |


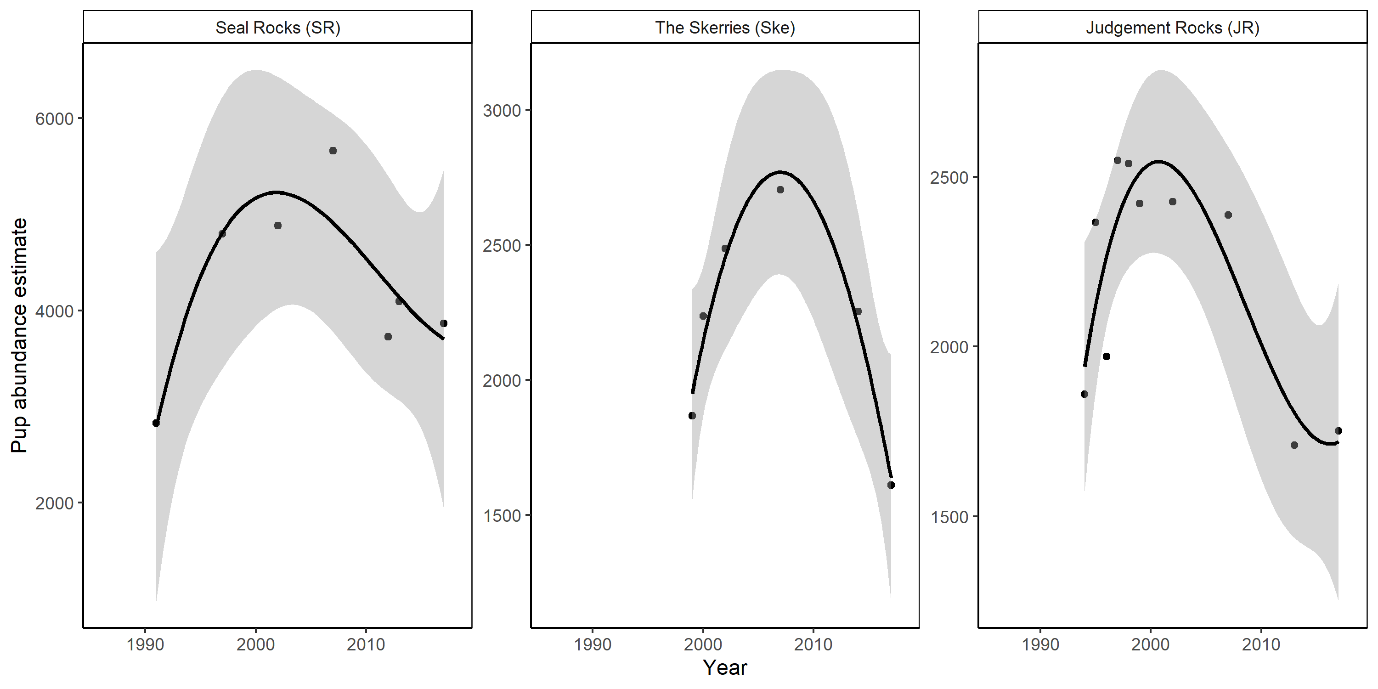


**S2 Fig. Smoothed predicted curves fitted to estimates of live pup abundance of Australian fur seal pups at three breeding colonies in south-eastern Australia, estimated using third-order polynomial linear model.** Live pup abundance estimates used at each site were determined by capture-mark-resight method.

**S3 Fig. Annual live pup counts at Seal Rocks from 2012 to 2017 are similar, as identified with labels.** The linear regression shown (dotted line) has an r^2^ of 0.392 and equation: y = -124.46x + 253627. Standard deviation is shown by error bars, which were small for 2012 because of count similarity (n=2), counts in other years had three to six independent replicates. Data for Seal Rocks in 2017 were sourced from [Sorrell et al. (2019)](#_ENREF_75) and all raw data are provided in S2 File.

**References**

Arnould JPY, Boyd IL, Warneke RM. 2003. Historical dynamics of the Australian fur seal population: evidence of regulation by man? Canadian Journal of Zoology 81:1428-1436. Department of Primary Industries P, Water and Environment. Fur Seal Census of Southwest Tasmania 26-28 February 2015. Internal Report. 2015. 17 pp.

Arnould JPY, Littnan CL. Pup production and breeding areas of Australian fur seals *Arctocephalus pusillus doriferus* at Kanowna Island and The Skerries in northeastern Bass Strait. Australian Mammalogy. 2000;22(1):51-5.

Goldsworthy SD, Bailleul F, Shaughnessy PD, Mackay AI, Reinhold S-L, Stonnill M, Lashmar K. 2016. Monitoring of pinniped populations on Kangaroo Island: 2015/16. Report to the Department of Environment, Water and Natural Resources, SARDI Publication No. F2014/000332-3, SARDI Research Report Series No. 901. Henley Beach, South Australia: SARDI Aquatic Sciences.

Goldsworthy SD, Bailleul F, Shaughnessy PD, Stonnill M, Lashmar K, Mackay AI. 2017. Monitoring of Seal Bay and other pinniped populations on Kangaroo Island: 2016/17. Report to the Department of Environment, Water and Natural Resources. South Australian Research and Development Institute (Aquatic Sciences), Adelaide.: SARDI Publication No. F2014/000322-4. SARDI Research Report Series No. 951. 40pp. Henley Beach, South Australia: SARDI Aquatic Sciences.

Kirkwood R, Gales R, Terauds A, Arnould JPY, Pemberton D, Shaughnessy PD, et al. Pup production and population trends of the Australian fur seal *(Arctocephalus pusillus doriferus)*. Marine Mammal Science. 2005;21(2):260-82.

Kirkwood R, Pemberton D, Gales R, Hoskins AJ, Mitchell A, Shaughnessy PD, et al. Continued population recovery by Australian fur seals. Marine and Freshwater Research. 2010;61:695-701.

Lewis CV. 1929. Report of the Chief Inspector of Fisheries and Game. Investigation into the feeding habits, etc., of seals in Victorian waters. Victoria. 17 p.

Le Souef AS. Notes on the seals found in Australian seas. Australian Zoologist. 1925;4:112-6, Plates XIII-XV.

Ling JK. Impact of colonial sealing on seal stocks around Australia, New Zealand and subantarctic islands between 150 and 170 degrees east. Australian Mammalogy. 2002;24:117-26.

Littnan CL, Mitchell AT. Australian and New Zealand fur seals at the Skerries, Victoria: recovery of a breeding colony. Australian Mammalogy. 2002;24:57-64.

McIntosh, R. R., S. P. Kirkman, S. Thalmann, D. R. Sutherland, A. Mitchell, J. P. Y. Arnould, M. Salton, D. J. Slip, P. Dann and R. Kirkwood (2018). Understanding meta-population trends of the Australian fur seal, with insights for adaptive monitoring. PLoS One 13(9): e0200253.

Pearse RJ. Distribution and conservation of the Australian fur seal in Tasmania. Victorian Naturalist. 1979;96:48-53.

Pemberton D, Gales R. Australian fur seals (*Arctocephalus pusillus doriferus*) breeding in Tasmania: population size and status. Wildlife Research. 2004;31(3):301-9.

Pemberton D, Kirkwood RJ. Pup production and distribution of the Australian fur seal, *Arctocephalus pusillus doriferus*, in Tasmania. Wildlife Research. 1994;21(3):341-52.

Shaughnessy PD, Briggs SV, Constable R. Observations on seals at Montague Island, New South Wales. Australian Mammalogy. 2001;23:1-7.

Shaughnessy PD, Goldsworthy SD. Abundance of fur seal pups on North Casuarina Island, South Australia in the 2011-12 breeding season. Report to Nature Foundation South Australia. South Australian Museum and South Australian Research and Development Institute, 2012.

Shaughnessy PD, Kirkwood RJ, Warneke RM. Australian fur seals, *Arctocephalus pusillus doriferus*: Pup numbers at Lady Julia Percy Island, Victoria, and a synthesis of the species' population status. Wildlife Research. 2002;29(2):185-92.

Shaughnessy PD, McKenzie J, Lancaster ML, Goldsworthy SD, Dennis TE. Australian fur seals establish haulout sites and a breeding colony in South Australia. Australian Journal of Zoology. 2010;58(2):94-103.

Shaughnessy PD, Testa JW, Warneke RM. Abundance of Australian fur seal pups, *Arctocephalus pusillus doriferus*, at Seal Rocks, Victoria, in 1991-92 from Petersen and Bayesian estimators. Wildlife Research. 1995;22(6):625-32.

Shaughnessy PD, Troy SK, Kirkwood R, Nicholls AO. Australian fur seals at Seal Rocks, Victoria: Pup abundance by mark-recapture estimation shows continued increase. Wildlife Research. 2000;27(6):629-33.

Shaughnessy PD, Warneke RM. Australian fur seal, *Arctocephalus pusillus doriferus*. In: Croxall JP, Gentry RL, editors. Status, biology, and ecology of fur seals Proceedings of an International Symposium and Workshop, Cambridge, England, 23-27 April 1984. NOAA Technical Report NMFS 51: US Dept. Commer.; 1987. p. 73-7.

Sorrell KJ, Clarke RH, Holmberg R, McIntosh RR. Remotely piloted aircraft improve precision of capture-mark-resight population estimates of Australian fur seals. Ecosphere. 2019;10(8):e02812. 10.1002/ecs2.2812.

Stamation KA, Shaughnessy PD, Constable AJ. Status of Australian fur seals, *Arctocephalus pusillus doriferus* (Carnivora: Otariidae) at Cape Bridgewater, Victoria. Australian Mammalogy. 1997;20(1):63-70.

Stockton J. Seals in Tasmanian prehistory. Proceedings of the Royal Society of Victoria. 1982;94:53-60.

Warneke RM. Seals of Westernport. Wildlife Circular No. 18. Victoria's Resources. 1966;8(2):4 p.

Warneke RM. Australian Fur Seal. Mammals in the seas. Volume 2: Pinniped species summaries and report on sirenians: FAO Fisheries Series; No. 5, Vol. 2.; 1979. p. 41-4.

Warneke RM. Report on an aerial survey of Australian fur seal sites in Victoria and Tasmania during the 1986 breeding season. Canberra Australian National Parks and Wildlife Service, 1988.

Warneke RM. The distribution and abundance of seals in the Australasian region, with summaries of biology and current research. Mammals in the seas, FAO fisheries series No5, Volume 4. IV. Rome: FAO; 1982. p. 431-75.

Warneke RM, Shaughnessy PD. A*rctocephalus pusillus*. The South African and Australian fur seal: Taxonomy, evolution, biogeography, and life history. In: Ling JK, Bryden MM, editors. Studies of sea mammals in south latitudes. Adelaide: South Australian Museum; 1985. p. 53-77.
